# Une localisation inhabituelle d’un carcinome primitif cutané rare: à propos d’un cas

**DOI:** 10.11604/pamj.2022.41.329.28965

**Published:** 2022-04-22

**Authors:** Layla Tahiri Elousrouti, Rafik Bentayeb, Amal Douida, Hakima Abid, Mohamed Abraki, Adil Ibrahimi, Sara Elloudi, Hanane Baybay, Fatimazahra Elmernissi, Youssef Alaoui Lamrani, Nawal Hammas, Hinde Elfatemi, Laila Chbani

**Affiliations:** 1 Laboratoire d´Anatomie et de Cytologie Pathologiques, Centre Hospitalier Universitaire Hassan II de Fès, Fès, Maroc,; 2 Laboratoire de Recherche Biomédicale et Translationnelle, Faculté de Médecine et de Pharmacie, Université Sidi Mohammed Ben Abdellah, Fès, Maroc,; 3 Service de Gastro-entéro-hépatologie, Centre Hospitalier Universitaire Hassan II de Fès, Fès, Maroc,; 4 Service de Dermatologie, Centre Hospitalier Universitaire Hassan II de Fès, Fès, Maroc,; 5 Service de Radiologie, Centre Hospitalier Universitaire Hassan II de Fès, Fès, Maroc

**Keywords:** Carcinome scléreux eccrine, carcinome annexiel microkystique, immunohistochimie, péri-anal, cas clinique, Eccrine sclerosus carcinoma, microcystic annexal carcinoma, immunohistochemistry, perianal, case report

## Abstract

Résumé

Les carcinomes annexiels sont rares et représentent moins de 1% des carcinomes cutanées. Le carcinome scléreux des glandes sudorales a été décrit pour la première fois en 1982 par Goldstein et al. Nous rapportons un nouveau cas de localisation inhabituelle péri. Il s´agit d´une patiente de 33 ans, présentait une lésion cutanée péri-anale, d´aspect rétracté. L´analyse histologique de la biopsie cutanée péri-lésionnelle, l´immunohistochimie, la négativité des investigations cliniques, radiologiques et endoscopiques nous ont permis de poser le diagnostic du carcinome scléreux eccrine. Il s´agit d´une entité rare, de localisation habituelle faciale, d´évolution lente mais agressive. Elle pose le problème de diagnostic différentiel avec des tumeurs bénignes et malignes d´où l´enjeu pour le pathologiste de savoir évoquer ce carcinome devant toute lésion cutanée d´aspect scléreux et infiltrant, d´évolution lente et dans un contexte de conservation de l´état général et d´absence d´histoire néoplasique, et de ne pas hésiter à demander de nouvelles biopsies profondes si doute.

## Abstract (en)

Adnexal carcinomas are rare, accounting for less than 1% of skin carcinomas. Sclerosus carcinoma of the sweat glands was first described by Goldstein et al. in 1982. We here report the case of a 33-year-old female patient with a retracted perianal skin lesion. Histological examination of perilesional skin biopsy, immunohistochemistry, and negative results of laboratory tests, radiological and endoscopic investigations allowed for the diagnosis of eccrine sclerosus carcinoma. This is a rare tumor, usually characterized by facial localization and slow but aggressive progression. It poses problems in differential diagnosis with benign and malignant tumors; hence the challenge encountered by pathologist of suspecting this carcinoma in patients with any sclerotic and infiltrating skin lesion characterized by slow progression, in a context of preservation of the general state and in the absence of neoplastic history as well as of feeling free to ask for new deep biopsies when in doubt.

## Introduction

Les carcinomes annexiels sont rares et représentent moins de 1% des tumeurs malignes cutanées [1]. Le carcinome scléreux des glandes sudorales, également nommé carcinome annexiel microkystique, a été décrit pour la première fois en 1982 par Goldstein *et al*. [2]. En absence d´arguments histologiques et immunohistochimique, ce diagnostic pose le problème de diagnostic différentiel avec l´adénocarcinome eccrine NOS qui reste un diagnostic d´élimination. Elle pose également le problème de diagnostic différentiel avec des tumeurs bénignes et d´autres tumeurs malignes d´où l´enjeu pour le pathologiste de savoir évoquer ce diagnostic. Nous rapportons un nouveau cas de localisation inhabituelle péri anale chez une jeune patiente en bon état général.

## Patient et observation

**Information du patient:** une femme de 33 ans, femme au foyer, sans antécédents particuliers, qui présentait depuis un an un prurit péri-anal mise sous traitement symptomatique sans amélioration.

**Résultats cliniques:** à l´examen physique, on trouvait une performance status à 0, un bilan étiologique de prurit est réalisé, revenant normal. Par la suite, la symptomatologie s´est aggravée par l´apparition d´une induration péri-anale qui s´étend à tout le périnée avec épaississement scléreux cutané. Il n´est pas noté d´autres signes associés ni d´altération de l´état général.

**Chronologie:** l´histoire de la maladie remonte à un an par l´apparition d´un prurit péri anal motivant la consultation chez plusieurs médecins qui l´ont mis sous traitement symptomatique sans amélioration, puis un bilan de prurit a été demandé, revenu normal. Par la suite un dermatologue a procédé à une biopsie cutanée.

**Figure 1:**
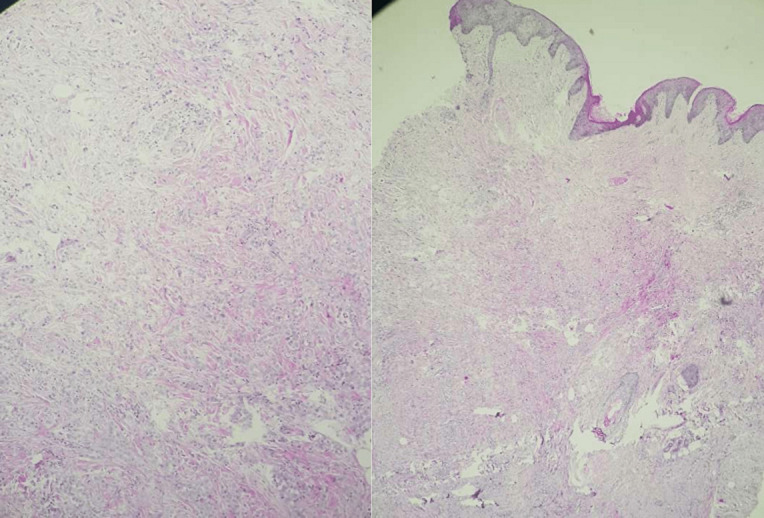
tissu cutané dont le derme et l´hypoderme sont infiltrés par une prolifération tumorale (a: HES x 40, b: HES x200)

**Figure 2:**
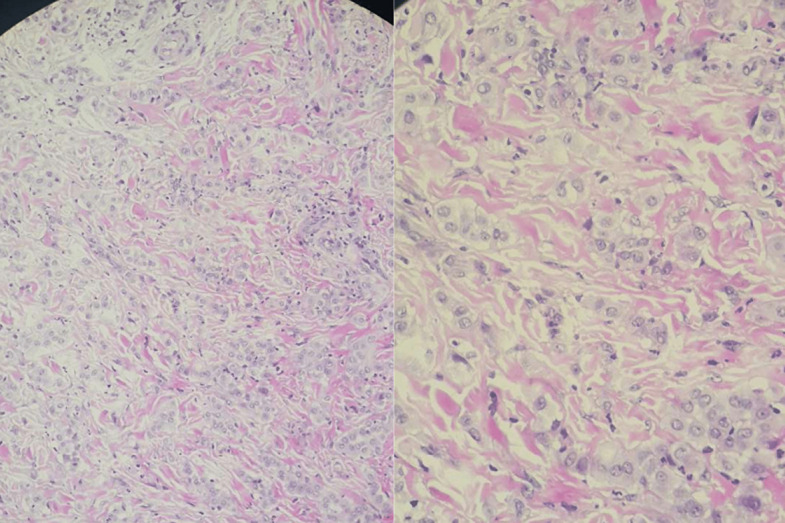
les cellules sont de grandes tailles, munies de noyaux irréguliers, tantôt hyperchromatiques tantôt à chromatine vésiculeuse et nucléolés; le cytoplasme est éosinophile (HES x 400)

**Figure 3:**
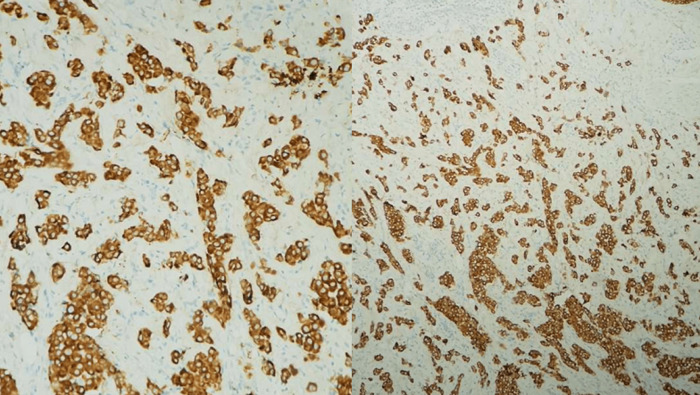
expression de l´anticorps anti- CKAE1/A3E3 par les cellules tumorales

**Démarche diagnostique:** une biopsie de la peau péri-anale est réalisée suspectant un lichen scléro-atrophique ou une Morphée. L´examen histologique retrouve un tissu cutané tapissé par un épiderme régulier, le derme et l´hypoderme sont manifestement infiltrés par une prolifération tumorale maligne, disposée en amas, en travées grêles et en rares structures glandulaire. Les cellules sont de grandes tailles, munies de noyaux irréguliers, tantôt hyperchromatiques tantôt à chromatine vésiculeuse et nucléolés. Le cytoplasme est éosinophile. Quelques figures de mitoses sont observées. Le stroma est scléreux et comporte des engainements périnerveux (Figure 1, Figure 2, Figure 3). Il n´est pas noté de différenciation malpighienne sur ce prélèvement.

**Figure 4:**
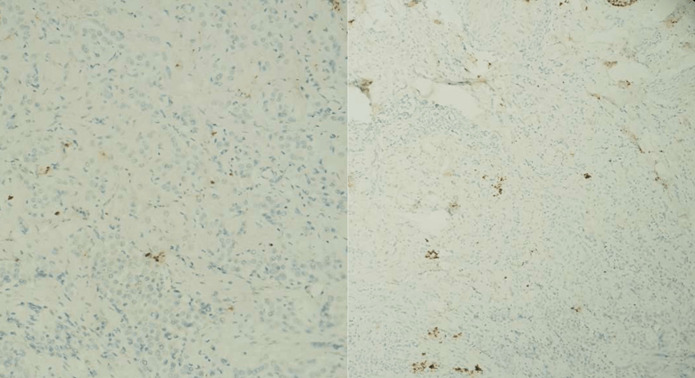
expression de l’anticorps anti- CK7 par les cellules tumorales

**Figure 5:**
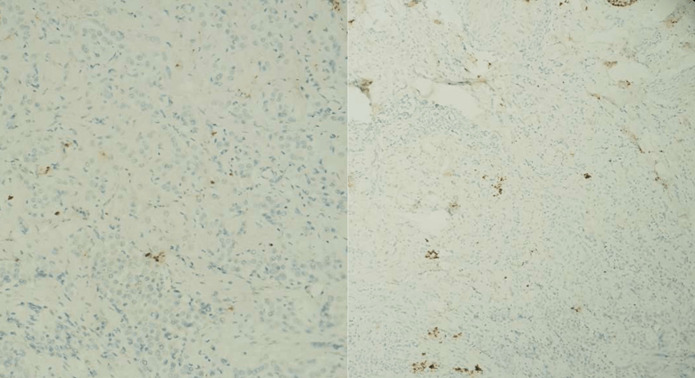
absence d’expression des anticorps anti- P63, anti-P40, anti-CK20 et anti-CDx2 par les cellules tumorales

**Figure 6:**
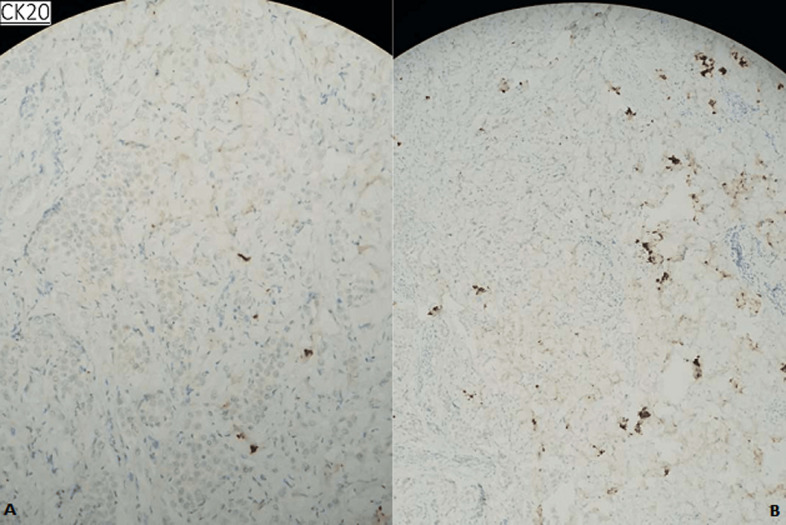
a) infiltration de la paroi rectale par le processus carcinomateux (HES x 200); b) immunohistochimie: expression de l’anticorps anti-CK7

**Figure 7:**
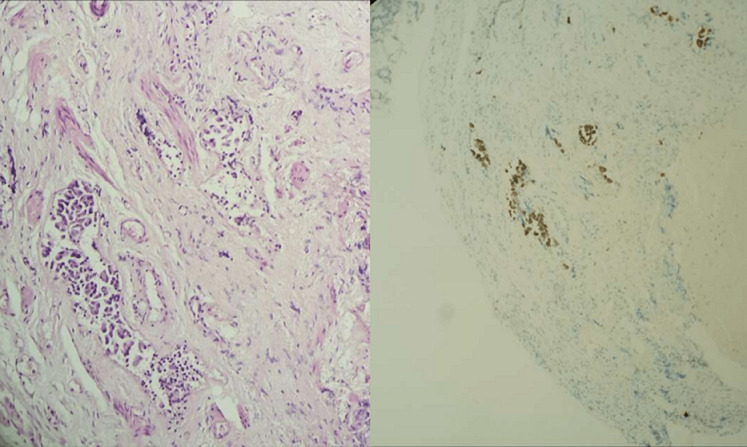
infiltration de la paroi rectale par le processus carcinomateux CK7+

A l´étude immunohistochimique, les cellules tumorales expriment la CK AE1/AE3, la CK7. Elles n´expriment pas l´Ecadhérine, la P63, la CK20, le CDX2, l´Her2, les récepteurs hormonaux, les marqueurs mélaniques, les marqueurs neuroendocrines ni les marqueurs lymphoïdes (Figure 4, Figure 5, Figure 6). Une localisation cutanée d´un carcinome peu différencié a été évoquée. Un bilan d´extension est demandé afin d´étiqueter l´origine (examen, gynécologique (avec FCV et biopsie du col), examen proctologique, une TDM thoraco-abdomino-pelvienne, une endoscopie digestive haute et basse (avec des biopsies étagées) et une écho-mammographie mammaire), tout est revenu normal en dehors de la TDM qui objective un épaississement de la graisse péri-rectale et anale, ainsi que le grand ementum et le mésentère. Les marqueurs tumoraux (ACE et CA19-9) sont négatifs. Par la suite, une IRM périnéo-pelvienne est réalisée objectivant les mêmes atteintes décrites sur la TDM ainsi qu´un envahissement des plans musculaires périnéaux et du rectum (confirmé par l´histologie de la biopsie rectale) (Figure 7).

**Intervention thérapeutique:** une radiothérapie est indiquée après discussion en réunion de concertation pluridisciplinaire, vu l´étendue de la lésion, et l´impossibilité de subir une intervention chirurgicale carcinologique.

**Suivi et résultats des interventions thérapeutiques:** après la fin de la radiothérapie, une IRM d´évaluation a été réalisée, objectivant une bonne réponse radiologique.

**Consentement éclairé:** la patiente a donné son consentement éclairé.

## Discussion

Le carcinome scléreux des glandes sudorales a été décrit pour la première fois en 1982 par Goldstein *et al*. [2], ils ont décrit dans leur papier cinq tumeurs siégeant au niveau du visage et se caractérisent histologiquement par une différenciation glandulaire eccrine, des kystes kératosiques, des cordons et des amas cellulaires uniformes, une stroma collagenique et scléreuse, une infiltration des tissus sous cutanés et une invasion périnerveuse. Par la suite, en 1985, Cooper et al. ont revu une série des 2000 cas de carcinomes cutanés de localisation faciale, à partir de laquelle ils ont sélectionné 20 cas, dont 3 pathologistes ont été d´accord pour le diagnostic de carcinome scléreux des glandes sudorales [3]. Ce groupe de carcinome annexiel, qui partage les aspects morphologiques sus cités, se caractérise par sa rareté et son évolution clinique lente et profonde, parfois sur des années, ce qui lui confère une présentation clinique bénigne sous forme de plaques, papules, ou indurations dont l´épiderme est souvent normale ou rétractée. Ceci pourrait conduire à un retard de diagnostic de malignité entraînant ainsi un retard de prise en charge optimale de la tumeur et une augmentation de la morbidité en raison d´une infiltration profonde et progression localement avancé comme l´illustre notre cas [4].

Cette tumeur survient chez l´adulte jeune et le sujet âgé avec un âge moyen de 44-64 ans, sans prédilection de sexe [5]. Dans 85% des cas, la tumeur se développe au niveau de la région tête et cou, avec une préférence pour la peau périorbitaire et la région centro-faciale [6], des cas ont été rapporté au niveau de la région axillaire, des membres, du tronc, des fesses et de la région génitale dont la vulve [7] et la région péri-anal qui a été décrite pour la première fois par Murata et al. en 1997 [8] et depuis aucun cas n´a été rapporté dans cette localisation à notre connaissance, notre cas est considéré le deuxième. L´antécédent lointain d´exposition aux rayonnements radioactifs est retrouvé chez environ 19%-50% des patients atteints de ce carcinome, d´autres facteurs prédisposant comprennent l´exposition aux rayons ultraviolets et l´immunodépression [9]. Ce carcinome a porté plusieurs dénominations depuis sa première description, dans la 4^e^ édition de la classification OMS des tumeurs de la peau 2018, il est appelé « carcinome annexiel microkystique » et ayant comme synonymes « carcinome scléreux des glandes eccrines » [10].

Il s´agit d´une tumeur dermique à caractère invasif avec un tropisme nerveux. Elle est faite de proportions variables de travées, de massifs essentiellement en profondeur et en zones d´invasion périlésionnelles, des glandes étirées et des kystes kératinisants sont observés essentiellement en superficie, la présence des deux aspects architecturaux permet de poser le diagnostic. Les cellules s´organisent au moins en deux assises, elles sont monomorphes, basophiles, au noyau arrondi, à chromatine dense ou vésiculeuse, au cytoplasme réduit, les mitoses sont rares. La nécrose est habituellement absente. Les engainements périnerveux sont classiques. Le stroma est scléreuse [1, 9, 10]. Dans les localisations habituelles (visage), le diagnostic différentiel se fait essentiellement avec le carcinome basocellulaire sclérodermiforme, le trichoépithéiome desmoplastique, et le syringome, le diagnostic est autant difficile si la biopsie est superficielle. La présence des kystes kératinisants est une aide diagnostique. Le syringome se représente sous forme de petites papules multiples et superficielles alors que le carcinome scléreux eccrine se présente sous forme de lésion unique, rétractée et invasive [9, 10].

Dans les localisations inhabituelles, comme l´est notre cas, le diagnostic différentiel se fait avec une métastase d´un carcinome d´une autre origine, dans ce cas l´histoire clinique et l´immunohistochimie permettent de redresser le diagnostic. La négativité du BEREP4 est un marqueur fiable pour éliminer le CBC sclérodermiforme (BEREP4+). Les marqueurs de cellules souches folliculaires CK15, CK19, sont utilisés pour discriminer le trichoépithéliome desmoplastique du carcinome scléreux eccrine. Ce dernier est CK19+/CK15-, à l´inverse du trichoépithéliome desmoplastique [11]. L´EMA et l´ACE soulignent les structures glandulaires sébacées. La p63 a une positivité hétérogène avec un marquage uniquement des cellules périphériques des massifs tumoraux dans le carcinome scléreux eccrine [12]. La chirurgie radicale avec marges saines, notamment la microchirurgie de Mohs dans les localisations faciales péri-orificielles, reste le traitement de référence. La radiothérapie est indiquée en adjuvant dans les cas à haut risque de récidive ou dans les cas dont la chirurgie est impossible comme notre cas. La chimiothérapie et les thérapeutiques ciblées sont en cours d´étude [13]. La surveillance doit être rapprochée tous les 6 à 12 mois pendant les 5 premières années après traitement en raison de risque de récidive allant de 15 à 60% [13].

## Conclusion

Ce cas illustre une localisation inhabituelle d´un carcinome annexiel cutané rare. Il faut penser au carcinome annexiel microkystique ou carcinome scléreux eccrine devant toute lésion cutanée d´aspect clinique scléreux rétracté, d´évolution lente et dans un contexte de conservation de l´état général et d´absence d´histoire clinique néoplasique. Une biopsie assez profonde est essentielle pour faire un bon diagnostic anatomopathologique et une meilleure prise en charge thérapeutique.
